# A13 PRECISION PHAGE THERAPY MITIGATES COLITIS AND ENHANCES STEROID EFFICACY THROUGH SUPPRESSION OF CROHN’S-ASSOCIATED BACTERIAL VIRULENCE

**DOI:** 10.1093/jcag/gwaf042.013

**Published:** 2026-02-13

**Authors:** K Jackson, H Galipeau, A Hann, M Zangara, M Bording-Jorgensen, B Coombes, Z Hosseinidoust, E F Verdu

**Affiliations:** McMaster University Faculty of Health Sciences, Hamilton, ON, Canada; McMaster University Faculty of Health Sciences, Hamilton, ON, Canada; McMaster University Faculty of Health Sciences, Hamilton, ON, Canada; McMaster University Faculty of Health Sciences, Hamilton, ON, Canada; McMaster University Faculty of Health Sciences, Hamilton, ON, Canada; McMaster University Faculty of Health Sciences, Hamilton, ON, Canada; Medicine, McMaster University Faculty of Engineering, Hamilton, ON, Canada; McMaster University Faculty of Health Sciences, Hamilton, ON, Canada

## Abstract

**Background:**

Current inflammatory bowel disease (IBD) therapies neglect microbial drivers. Treatment failures lead to dose escalation and risk of adverse effects. Pathobiont bacteria, like adherent-invasive *Escherichia coli* (AIEC), have been postulated as a microbial driver in IBD, however, their targeting remains elusive for antibiotics are non-specific and disturb the background microbiome. Attention has shifted towards bacteriophages, yet their underlying mechanisms and adjunctive therapy capabilities, remain under-investigated.

**Aims:**

We assess whether and how phage, specific for a CD-associated bacterium, reduces colitis severity using gnotobiotic mouse models.

**Methods:**

*In vitro* kill curves and biofilm challenges identified lytic phage. Germ-free C57BL/6 mice were colonized with altered Schaedler-flora (ASF) and *E. coli* NRG857c (NRG). Mice were treated with phage (1x10^9^ PFU/dose; daily or TIW) or vehicle (PBS) for 2 weeks (n = 6/group). Dextran sulfate sodium (2%; DSS) in drinking water induced colitis. Phage treatment was also tested in a spontaneous model of colitis using C57BL/6NTac-*Il10*^*em8Tac*^ (IL-10^-/-^) mice, treated weekly. Mice were monitored daily for weight, stool consistency, and occult blood. Fecal bacterial load was determined, and the orientation of *fimS* was quantified using qPCR to assess virulence. Endpoint histological and immunohistochemistry analysis were performed to quantify colitis severity and NRG infiltration, respectively. Additional colonized C57BL/6 mice received a low-dose of budesonide (2ug/day) (n = 5) or vehicle (n = 5) until endpoint. Mice were monitored daily for disease activity and colon tissues were collected for histological analysis. HPLC was used to determine active budesonide levels.

**Results:**

HER259 was identified as a lytic phage against NRG. In colonized mice, HER259 reduced clinical symptoms (p < 0.001, p < 0.001) and histological scores (p < 0.0001, p < 0.001) in induced and spontaneous colitis, respectively. A 1-log reduction in bacterial load was observed in HER259-treated mice. Reisolated NRG exhibited a reduced FimS ON/OFF ratio (p < 0.01). Immunohistochemistry revealed reduced infiltration of NRG into the lamina propria following HER259 treatment. HER259 treatment increased the efficacy of a sub-therapeutic dosing regimen of budesonide (p < 0.001), which was independent of microbial-based drug metabolism as determined by HPLC.

**Conclusions:**

We demonstrate that lytic phage targeting a CD-associated bacterium attenuates colitis in gnotobiotic mice. Intervention did not lead to bacterial eradication but instead modulated bacterial virulence mechanisms and enhanced responses to corticosteroid therapy. The data suggest alternative mechanisms should be considered when translating phage therapy to treat IBD in clinical trials.

**Funding Agencies:**

CIHR

